# High-Resolution 3T Intracranial Vessel Wall MRI in Patients Evaluated for Suspected Inflammatory Intracranial Vasculopathy: Morphologic and Follow-Up Findings from a Retrospective Case Series

**DOI:** 10.3390/tomography12080116

**Published:** 2026-08-19

**Authors:** Yeliz Basar, Mujgan Orman, Tugce Ozdemir Gultekin, Ercan Karaarslan, Civan Islak

**Affiliations:** 1 Department of Radiology, Acibadem Healthcare Group, 34457 Istanbul, Turkey; 2 Department of Neurology, Acibadem Healthcare Group, 34457 Istanbul, Turkey; 3 Department of Radiology, School of Medicine, Mehmet Ali Aydinlar Acibadem University, 34638 Istanbul, Turkey; 4 Department of Radiology, Memorial Sisli Hospital, 34385 Istanbul, Turkey

**Keywords:** central nervous system vasculitis, primary angiitis of the central nervous system, vessel wall imaging, intracranial vasculopathy

## Abstract

Objective: Our objective was to describe morphologic features and longitudinal changes observed on high-resolution vessel wall imaging (HR-VWI) in a selected retrospective case series of patients evaluated for suspected inflammatory intracranial vasculopathy. Methods: This retrospective case series included patients who underwent 3T intracranial HR-VWI for suspected inflammatory intracranial vasculopathy. Clinical and imaging data were collected from medical records and imaging archives. Three radiologists reviewed baseline and follow-up examinations for vessel wall enhancement (VWE) pattern and grade, wall thickness, luminal narrowing, arterial distribution, diffusion-restricted lesions, and susceptibility-sensitive findings. Analyses were descriptive and patient-level. Each patient contributed one baseline and one final examination to longitudinal summaries; intermediate scans characterized individual trajectories only. Results: Nineteen patients were included, and 17 underwent serial HR-VWI. Median age was 46 years (IQR, 35.5–60.0 years), and 11 patients were female. Follow-up HR-VWI was available in 17 patients over a median of 391 days (IQR, 237–1819 days; range, 15–4034 days). VWE was present in all patients, with a purely concentric pattern in 15/19 (78.9%). The middle cerebral and internal carotid arteries were involved in 17/19 and 13/19 patients, respectively. Among the 17 patients with paired examinations, VWE grade decreased in 13 patients (76.5%) and remained stable in four patients (23.5%), with the median decreasing from 3.0 to 1.0. Maximum wall thickness decreased in 16 patients and remained stable in one, with the median changing from 2.0 mm to 1.4 mm. Conclusions: In this selected retrospective case series of patients evaluated for suspected inflammatory intracranial vasculopathy, 3T HR-VWI commonly demonstrated concentric VWE and provided descriptive longitudinal information regarding enhancement evolution. These findings should not be interpreted as evidence of diagnostic performance.

## Simple Summary

Inflammation of blood vessels in the brain can cause stroke and other serious neurological problems, but diagnosis is difficult. This study reviewed detailed vessel wall magnetic resonance imaging scans from 19 patients evaluated for possible inflammatory disease of the brain arteries; 17 had follow-up scans. Most patients showed circular enhancement of the artery wall, and enhancement and wall thickening decreased over time in many patients, while artery narrowing often remained stable. These findings show how vessel wall imaging can describe changes over time, but they do not prove that the imaging pattern is specific for vasculitis.

## 1. Introduction

Central nervous system (CNS) vasculitis represents a severe and potentially devastating group of inflammatory vascular disorders that can be broadly categorized as primary angiitis of the central nervous system (PACNS) or secondary cerebral vasculitis [1,2]. PACNS is a rare idiopathic condition characterized by isolated inflammation and destruction of small- and medium-sized vessels confined to the brain, spinal cord, and leptomeninges, without evidence of systemic involvement [3,4]. In contrast, secondary cerebral vasculitis occurs as a neurological manifestation of systemic autoimmune, inflammatory, or infectious disorders, including systemic lupus erythematosus, Behçet’s disease, and antineutrophil cytoplasmic antibody-associated vasculitis [5,6].

The clinical diagnosis of CNS vasculitis remains challenging because the presentation is heterogeneous and nonspecific, with manifestations such as persistent headache, encephalopathy, cognitive or behavioral change, seizure, transient ischemic attack, ischemic stroke, or hemorrhagic stroke [1,7,8]. Differentiating PACNS from secondary vasculitis and from noninflammatory mimics is clinically important but difficult, because no single noninvasive biomarker can definitively establish the diagnosis. Accurate etiologic classification is essential, as treatment strategies differ substantially, ranging from management of an underlying systemic disorder to CNS-directed immunosuppressive therapy in selected patients [9,10].

Conventional vascular imaging techniques, including computed tomography angiography (CTA), magnetic resonance angiography (MRA), and digital subtraction angiography (DSA), primarily assess luminal abnormalities rather than direct vessel wall pathology. Although these techniques can demonstrate stenosis, occlusion, or multifocal luminal irregularity, they do not directly assess the arterial wall, where the primary inflammatory process occurs [11,12]. Moreover, luminal findings may be nonspecific and can overlap among central nervous system vasculitis, reversible cerebral vasoconstriction syndrome, intracranial atherosclerotic disease, dissection, and other vasculopathies. Prior studies have emphasized that conventional luminal imaging may therefore be insufficient for determining the underlying etiology of intracranial arterial abnormalities, particularly in nonocclusive or early-stage disease [13,14].

High-resolution vessel wall imaging (HR-VWI) with magnetic resonance imaging (MRI) has emerged as a useful adjunct to conventional luminal imaging by enabling direct visualization of arterial wall thickening and contrast enhancement. HR-VWI is increasingly used in the evaluation of intracranial vasculopathies, including inflammatory vasculopathies, intracranial atherosclerosis, reversible cerebral vasoconstriction syndrome, moyamoya vasculopathy, and dissection. In practical interpretation, smooth, homogeneous concentric wall thickening and enhancement involving one or more arterial segments are considered supportive of inflammatory vasculopathy. In contrast, eccentric plaque-like wall thickening favors intracranial atherosclerotic disease; an intimal flap or intramural hematoma favors arterial dissection; absent or mild wall enhancement together with reversibility of luminal narrowing on follow-up favors reversible cerebral vasoconstriction syndrome; and progressive terminal internal carotid artery narrowing with collateral formation favors moyamoya vasculopathy. However, substantial overlap exists among these patterns, and no individual HR-VWI feature is specific for CNS vasculitis. HR-VWI should therefore be interpreted together with clinical, laboratory, conventional MRI, luminal angiographic, and longitudinal findings [15,16,17,18]. Expert consensus recommendations from the American Society of Neuroradiology and the Society for Magnetic Resonance Angiography similarly position intracranial vessel wall MRI as an adjunct to conventional angiographic imaging and emphasize adequate blood-signal suppression and interpretation within the broader clinical and imaging context [17,18].

Despite increasing clinical use, data on HR-VWI in central nervous system vasculitis remain limited, particularly regarding real-world case series with longitudinal imaging. Descriptive institutional cohorts may therefore provide useful information about the spectrum of vessel wall findings, associated parenchymal abnormalities, and imaging changes over time. Accordingly, the aim of this retrospective descriptive case series was to characterize morphologic and longitudinal HR-VWI findings in a selected cohort of patients evaluated for suspected inflammatory intracranial vasculopathy, with emphasis on VWE pattern, arterial involvement, associated diffusion-restricted lesions, and available longitudinal imaging changes.

## 2. Materials and Methods

### 2.1. Study Design

**Figure 1:**
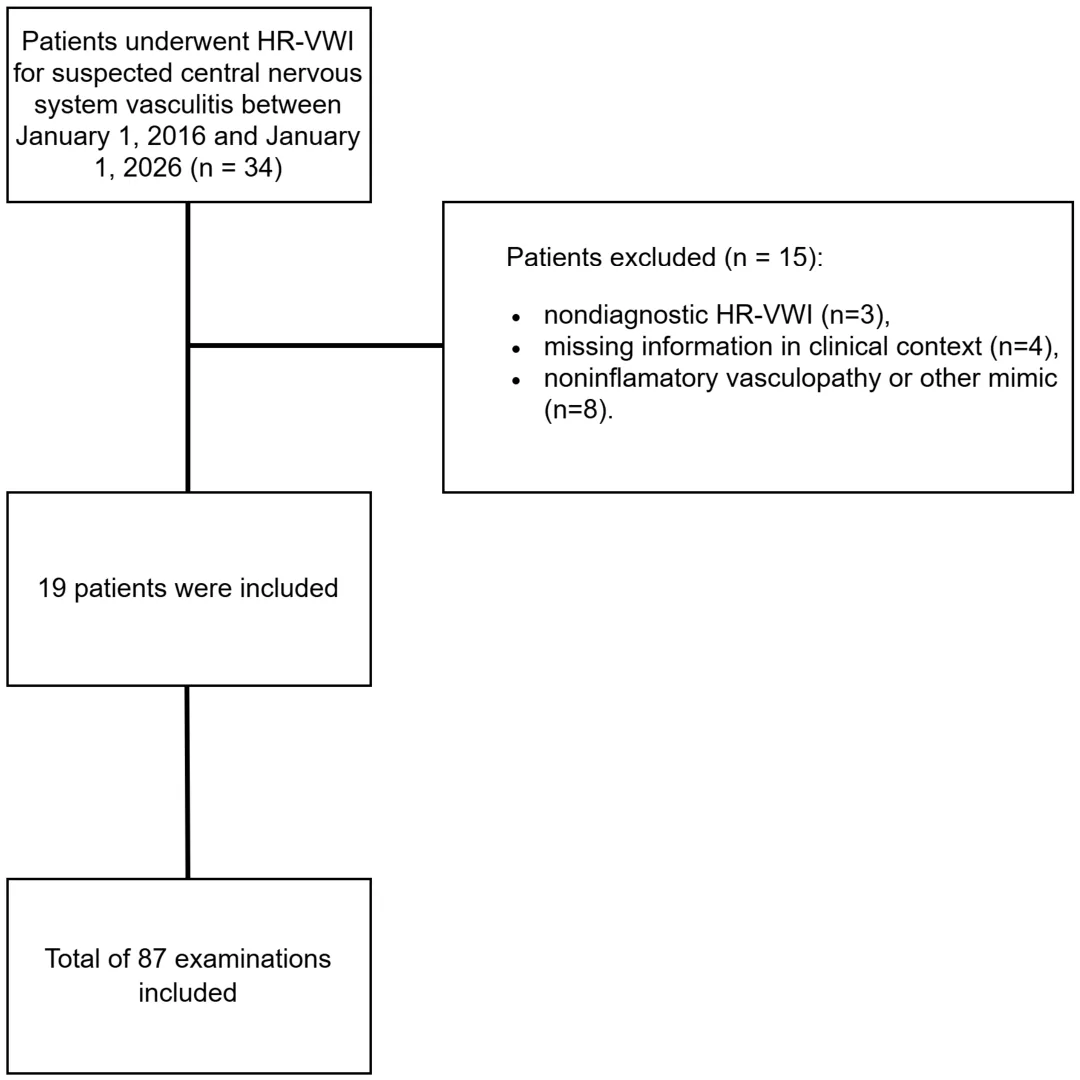
Flowchart of the study. HR-VWI: High-resolution vessel wall imaging.

The study was approved by the Acıbadem University and Acıbadem Healthcare Institutions Medical Research Ethics Committee on 26 March 2026 (approval no. 2026-06/236), and the requirement for informed consent was waived because of the retrospective design. The source HR-VWI examinations and clinical records covered the period from January 2016 through January 2026. Following ethics approval, the investigators retrospectively identified eligible cases, extracted and anonymized the data, reviewed the imaging examinations, and performed the analyses. No prospective recruitment or future data collection was undertaken. All unique patients who underwent intracranial HR-VWI for suspected inflammatory intracranial vasculopathy during the study period were identified from the institutional imaging archive. Thirty-four unique patients were assessed for eligibility. Fifteen patients were excluded because HR-VWI was nondiagnostic (*n* = 3), the available clinical information was insufficient for assessment (*n* = 4), or the multidisciplinary evaluation favored a noninflammatory vasculopathy or another alternative diagnosis (*n* = 8). The remaining 19 patients met the eligibility criteria and were included. Eligibility was not restricted by age. Patients younger than 18 years were retained because the study was designed to describe all eligible patients undergoing HR-VWI for suspected inflammatory intracranial vasculopathy during routine clinical care. The same HR-VWI acquisition and image-analysis framework was applied to pediatric and adult examinations. However, no uniform pediatric-specific diagnostic-confidence framework had been prospectively applied across the study period, and the available retrospective records did not permit reliable reassignment according to standardized childhood CNS vasculitis criteria. Pediatric diagnostic status therefore reflected the final multidisciplinary clinical assessment documented during routine care, as in the adult cases. Definite and probable diagnostic categories were not retrospectively assigned because standardized diagnostic-confidence criteria had not been prospectively applied to all patients. The diagnostic status reflected the final multidisciplinary assessment performed during clinical care. This assessment considered the clinical presentation, available laboratory and cerebrospinal fluid findings, conventional brain MRI, luminal angiographic findings, HR-VWI morphology, and longitudinal clinical or imaging evolution when available. Because the source records covered a long retrospective period, no uniform predefined diagnostic algorithm or minimum set of cerebrospinal fluid, serologic, angiographic, or histopathologic criteria had been applied across all patients. Clinical suspicion therefore reflected the final multidisciplinary assessment documented during routine clinical care rather than a retrospectively imposed diagnostic rule. Supporting diagnostic information was not uniformly available at the case level, and the available dataset did not permit reliable classification of patients as biopsy-confirmed, angiographically supported, or principally imaging-based. Alternative diagnoses were considered during routine clinical care when relevant information was available; however, systematic exclusion of every potential mimic could not be verified retrospectively. The patient selection process and study design are summarized in Figure 1. The study was designed as a descriptive clinical–radiological analysis of vessel wall MRI findings, associated parenchymal abnormalities, and available longitudinal imaging evolution. The study was reported in accordance with the STROBE recommendations.

### 2.2. Data Acquisition

Demographic and clinical variables were extracted from electronic medical records, including age, sex, presenting symptoms, vascular risk factors, established clinical diagnosis, diagnostic category, and available follow-up data.

### 2.3. MRI Protocol

All MRI examinations were performed on a 3T scanner (Siemens MAGNETOM Vida, Siemens Healthineers, Erlangen, Germany) using a dedicated head coil. The routine protocol included conventional brain MRI, intracranial angiographic imaging, and high-resolution intracranial VWI before and after intravenous gadolinium-based contrast administration. Conventional MRI sequences included, when available, axial T2-weighted imaging, fluid-attenuated inversion recovery imaging, diffusion-weighted imaging (DWI) with apparent diffusion coefficient maps, susceptibility-weighted or T2*-weighted imaging, pre- and postcontrast T1-weighted imaging, and time-of-flight MR angiography. For blood-signal suppression, the VWI protocol used a sagittal 3D variable-flip-angle turbo spin-echo SPACE fat-suppressed black-blood acquisition performed before and after contrast administration. The precontrast and postcontrast 3D T1 SPACE FS acquisitions were obtained using identical sequence parameters. Subtraction imaging was not performed; vessel wall enhancement was assessed by direct visual comparison of the matched precontrast and postcontrast images. Complete acquisition parameters are provided in Supplementary Table S1.

### 2.4. Image Analysis

All imaging examinations were reviewed using the institutional picture archiving and communication system by three radiologists (MO, YB and EK), blinded to final clinical diagnosis, with 10, 15 and 25 years of neuroimaging experience, respectively. All baseline and follow-up examinations were reviewed jointly by the three radiologists in consensus sessions rather than through independent assessments. Baseline and follow-up examinations were reviewed together, and the readers were aware of examination order. Formal independent readings, random ordering, reader blinding to examination order, and interobserver-agreement analysis were not performed. Treatment type and timing were not systematically available and were not formally incorporated into the imaging assessment. For each MRI examination, HR-VWI was evaluated for the presence, location, morphology, and intensity of vessel wall abnormality. The pattern of vessel wall thickening and enhancement was categorized as concentric, eccentric, mixed/asymmetric, or indeterminate. Concentric involvement was defined as circumferential wall thickening or enhancement involving the vessel wall symmetrically, whereas eccentric involvement was defined as asymmetric or focal wall abnormality involving only part of the vessel circumference. Potential physiologic arterial enhancement and venous contamination were evaluated by comparing matched precontrast and postcontrast images in multiple planes and across contiguous slices, with correlation to the corresponding artery on time-of-flight MR angiography or other available luminal imaging. Enhancement was attributed to the arterial wall only when it followed the expected arterial-wall contour and could be separated from an adjacent venous structure. Linear or tubular enhancement following a venous course, enhancement continuous with an identifiable vein, and equivocal enhancement that could not be confidently localized to the arterial wall were not classified as definite VWE. VWE was graded visually and semi-quantitatively on a 4-point scale: grade 0, no visible enhancement; grade 1, mild enhancement; grade 2, moderate enhancement; and grade 3, marked enhancement. Grading was based on consensus visual assessment of the arterial wall on precontrast and postcontrast HR-VWI. No fixed internal reference structure, including the pituitary stalk or venous enhancement, was used to calibrate enhancement intensity. Therefore, the grades represent ordinal visual categories rather than quantitative enhancement ratios. The maximum VWE grade and maximum vessel wall thickness were treated as patient-level, per-examination summary measures. For each examination, the maximum VWE grade was recorded from the most conspicuous enhancing arterial segment, and maximum wall thickness was measured at the site of greatest visible wall abnormality. The segment contributing the maximum value at follow-up was not required to be identical to the segment contributing the maximum value at baseline. Accordingly, the longitudinal comparisons represent changes in each patient’s maximum observed abnormality at the first and final evaluable examinations and should not be interpreted as segment-matched measurements of lesion regression. Images were not formally coregistered across examinations. Examination order was known because baseline and follow-up studies were reviewed together for longitudinal assessment. Treatment information was not systematically incorporated into the image review because treatment type and timing were incompletely available. Wall boundaries were identified visually on the available high-resolution images, and no dedicated correction for partial-volume effects or formal technical validation of measurement precision was performed. The reported thickness represents the maximum one-sided thickness of the visibly abnormal arterial wall, measured between the luminal and outer wall boundaries; it does not represent the total outer arterial diameter (Supplementary Figure S1). The arterial distribution of involvement was recorded according to the affected artery or arterial segment, including the intracranial internal carotid artery, anterior cerebral artery, middle cerebral artery, posterior cerebral artery, basilar artery, vertebral artery, posterior inferior cerebellar artery, and visible distal branches. Luminal imaging was reviewed for stenosis, occlusion, multifocal irregularity, or absence of visible luminal abnormality. Luminal narrowing was graded semi-quantitatively as grade 0, no narrowing; grade 1, mild narrowing; grade 2, moderate narrowing; and grade 3, severe narrowing or near-occlusion/occlusion. Parenchymal MRI was assessed for acute or early subacute ischemic lesions, chronic infarcts, FLAIR hyperintense lesions, hemorrhage or microbleeds on susceptibility-sensitive sequences, leptomeningeal enhancement, and parenchymal enhancement. An acute or early subacute ischemic lesion was defined as focal DWI hyperintensity with corresponding ADC reduction when present, interpreted together with lesion morphology and vascular distribution on conventional MRI. Because clinical stroke adjudication and exact symptom-to-imaging timing were not uniformly available, these findings were classified as MRI-defined ischemic lesions rather than clinically adjudicated infarctions. When diffusion-restricted lesions were present, their vascular territory was compared with the distribution of VWE. For patients with serial HR-VWI examinations, follow-up findings were categorized as complete resolution, partial regression, stable appearance, or progression of VWE compared with baseline. For each patient with serial HR-VWI, the primary follow-up interval was calculated as the number of days between the first and final evaluable HR-VWI examinations.

### 2.5. Statistical Analysis

All analyses were descriptive and were performed using Python 3.11 with SciPy (1.18.0) and statsmodels (0.14.6). Continuous variables are presented as medians with interquartile ranges and ranges, whereas categorical and ordinal variables are presented as counts and percentages. All analyses were conducted at the patient level. Longitudinal analyses were conducted at the patient level using the maximum abnormality recorded at each patient’s chronological first and final evaluable examinations. Because the arterial segment contributing the maximum value could differ between examinations, these analyses represent first-versus-final comparisons of patient-level maximum findings rather than segment-matched measurements. Follow-up intervals were categorized as ≤90 days, 91–365 days, 366–730 days, or >730 days. An adult-only descriptive sensitivity analysis was performed by excluding the four patients younger than 18 years. Because of the small group sizes, both analyses were descriptive, and no hypothesis testing was performed. Intermediate examinations were used only to characterize individual imaging trajectories and were not treated as independent observations. Variable-specific denominators were reported according to the availability of paired measurements. Changes in VWE grade, maximum wall thickness, and luminal narrowing grade were summarized using baseline and final medians and the numbers of patients showing a decrease, stable findings, or an increase. Missing data were not imputed, and the denominator for each summary reflects the available cases. Given the small sample size and retrospective case-series design, no formal hypothesis testing was performed.

## 3. Results

### 3.1. Study Cohort

The final study cohort comprised 19 patients who underwent 3T HR-VWI for suspected inflammatory intracranial vasculopathy. Serial HR-VWI was available in 17/19 patients, with a median follow-up interval of 391 days (interquartile range, 237–1819 days; range, 15–4034 days). Follow-up was ≤90 days in two patients, 91–365 days in six patients, 366–730 days in two patients, and >730 days in seven patients. Patient-level examination counts, follow-up durations, arterial involvement, and VWE patterns are provided in Supplementary Table S5.

The median patient age was 46 years (interquartile range, 35.5–60.0 years; range, 6–79 years). Eleven patients were female and eight were male. Four patients were younger than 18 years, including two 6-year-old patients, one 12-year-old patient, and one 15-year-old patient; the remaining 15 patients were adults. In the adult-only sensitivity analysis, 15 patients were included, of whom 13 had serial HR-VWI. VWE was present in all 15 adults; a purely concentric pattern was observed in 11/15 adults (73.3%), MCA involvement in 13/15 (86.7%), ICA involvement in 9/15 (60.0%), and MRI-defined acute or early subacute ischemic lesions in 10/15 (66.7%). Among the 13 adults with serial HR-VWI, VWE grade decreased in 10 patients (76.9%) and remained stable in three patients (23.1%); no increase was observed. Patient-level ages and clinical classifications are provided in Supplementary Tables S3 and S4. An established clinical diagnosis was present in 7/19 patients (36.8%), including Sjögren-associated vasculitis in two patients, nonspecified CNS vasculitis in two patients, primary CNS vasculitis in one patient, Takayasu arteritis in one patient, and rheumatoid arthritis-associated vasculitic involvement in one patient. The remaining 12/19 patients (63.2%) were included in the clinical-suspicion category because their overall clinical and imaging findings raised multidisciplinary suspicion for CNS vasculitis, but the diagnosis was not definitively confirmed. Because case-level CSF, serologic, angiographic, biopsy, and mimic-exclusion data were not uniformly documented in the available retrospective dataset, the patients could not be further classified reliably according to biopsy confirmation, angiographic support, or a principally imaging-based diagnosis. Both patients with an established clinical diagnosis and patients with clinical suspicion without definitive confirmation were retained to describe the HR-VWI findings encountered in this selected clinical cohort. Because the individual etiologic groups included only one or two patients, subgroup comparisons according to diagnosis were not performed. All 19 patients received treatment during their clinical course. However, treatment type, dose, duration, exact timing relative to HR-VWI examinations, treatment modifications, clinical response, relapse status, and disability outcomes could not be consistently retrieved from the retrospective records. Case-level summary was given in Table 1. Consequently, treatment–imaging and clinical–imaging correlations were not performed.

### 3.2. HR-VWI Findings

**Figure 2:**
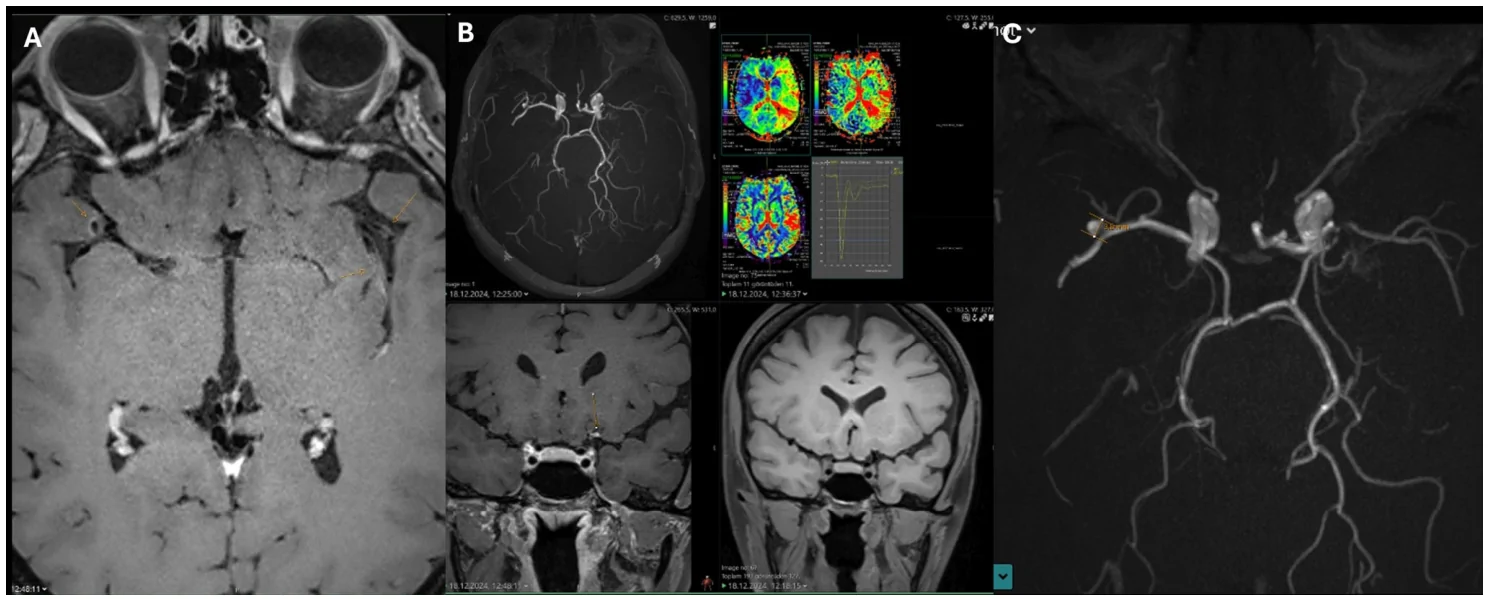
Asymmetric vessel wall enhancement with hemodynamic significance in a patient evaluated for suspected inflammatory intracranial vasculopathy. A 40-year-old woman evaluated for suspected inflammatory intracranial vasculopathy. (**A**) Vessel wall MRI shows asymmetric wall thickening and marked enhancement at the left ICA bifurcation and proximal left MCA. (**B**) Luminal imaging demonstrates severe proximal left MCA stenosis. (**C**) Perfusion imaging shows an approximately 2-s time-to-peak delay across the left MCA territory compared with the right hemisphere, suggesting hemodynamically significant involvement. A 4 × 3 mm saccular aneurysm is additionally present at the right MCA bifurcation.

VWE was identified in all included patients (19/19, 100%). A purely concentric enhancement pattern was observed in 15/19 patients (78.9%), whereas the remaining four patients showed mixed or asymmetric features. A representative example of asymmetric vessel wall enhancement with associated severe stenosis and perfusion delay is shown in Figure 2. Among the 19 patients, the median maximum VWE grade was 3 (IQR, 2–3): grade 3 was recorded in 11 patients, grade 2 in seven patients, and grade 1 in one patient.

The median maximum recorded vessel wall thickness across all 19 patients was 2.2 mm (IQR, 1.8–2.8 mm; range, 1.4–3.8 mm). Wall thickness measurements were obtained from the most abnormal vessel wall segment and generally corresponded to segments demonstrating VWE.

The most frequently involved arteries were the middle cerebral artery (17/19 cases) and internal carotid artery (13/19 cases), followed by the posterior cerebral artery (6/19 cases), vertebral artery (5/19 cases), basilar artery (3/19 cases), and posterior inferior cerebellar artery (2/19 cases). One case had extracranial common carotid/external carotid involvement recorded in the context of systemic large-vessel disease.

Luminal narrowing was variably present. Based on the case-level maximum luminal narrowing grade across all 19 cases, stenosis was grade 0 in three cases, grade 1 in six cases, grade 2 in four cases, and grade 3 in six cases; no case had missing or nonstandard stenosis grading.

### 3.3. Parenchymal MRI Findings

MRI-defined acute or early subacute ischemic lesions were present in 13/19 patients (68.4%). Chronic ischemic or post-ischemic parenchymal changes, including chronic infarcts and encephalomalacic changes, were recorded in several cases. Nonspecific FLAIR white matter hyperintensities were variably present, with available Fazekas grades ranging from absent white matter disease to grade 3 changes. Susceptibility-sensitive imaging demonstrated microbleeds in 9/19 cases (47.4%). Concomitant aneurysms were recorded in five cases, including previously treated or coil-embolized aneurysms. Additional chronic or associated imaging findings included chronic infarct and encephalomalacia in 14 cases, moyamoya-like collateralization around stenotic segments in two cases, Wallerian degeneration in two cases, an organized hematoma extending into the ventricle with late acute-to-early subacute hemorrhagic features in one case, intra- and extraconal orbital inflammation in one case, and chronic intraluminal thrombus at the level of occlusion in one case.

Median maximum luminal narrowing grade was 2.0 (IQR, 1.0–3.0) among patients with inferred acute or subacute infarction and 1.5 (IQR, 1.0–2.8) among patients without or with unclear acute or subacute infarction. Given the small cohort and descriptive study design, no formal association testing was performed, and these values should not be interpreted as establishing or excluding a relationship between luminal narrowing severity and infarction.

### 3.4. Follow-Up Vessel Wall MRI Findings

**Figure 3:**
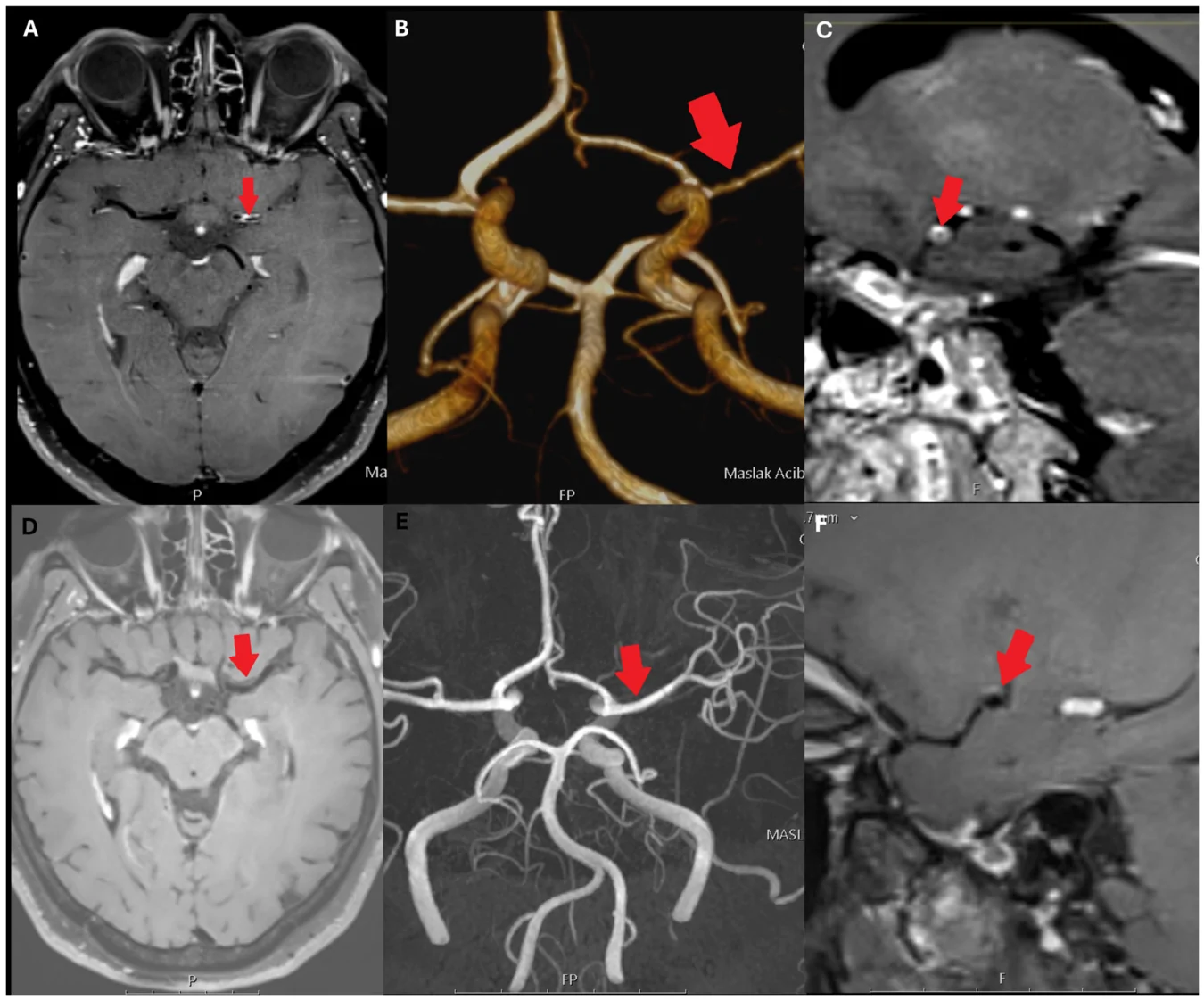
Longitudinal HR-VWI findings in a 42-year-old woman evaluated for suspected inflammatory intracranial vasculopathy. Arrows in panels (**A**,**C**,**D**,**F**) indicate the involved enhancing vessel wall, whereas the arrows in panels (**B**,**E**) indicate the corresponding luminal narrowing.

**Figure 4:**
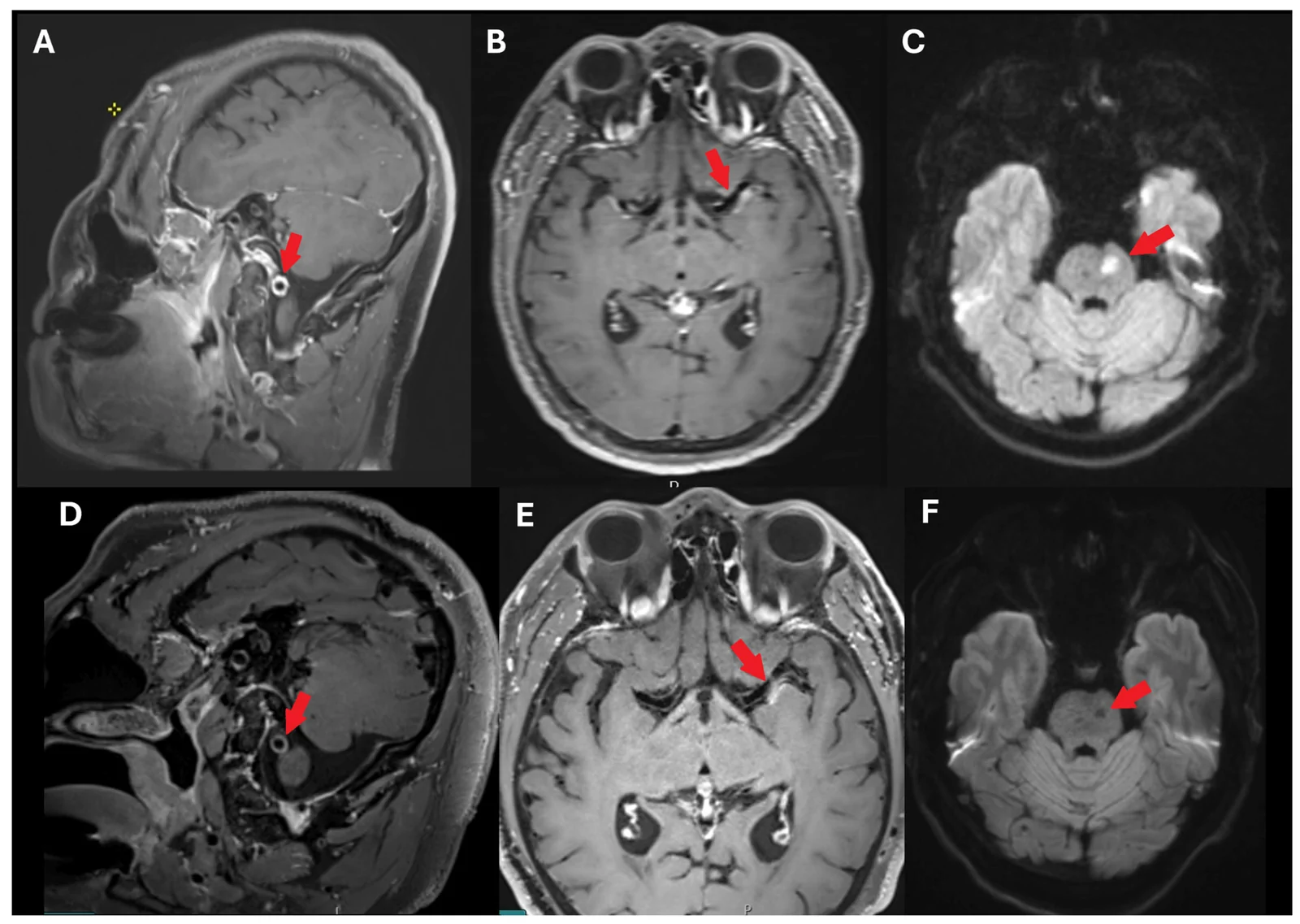
Multifocal baseline and follow-up HR-VWI findings in a 39-year-old man presenting with headache and transient ischemic attacks. Pretreatment postcontrast HR-VWI demonstrates concentric wall thickening and enhancement of the right vertebral artery V4 segment on the sagittal image ((**A**), arrow) and bilateral MCA M1 segments, more prominent on the left, on the axial image ((**B**), arrow). Diffusion-weighted imaging shows a 12-mm acute diffusion-restricted infarct in the left paramedian pons ((**C**), arrow). Follow-up postcontrast HR-VWI after treatment demonstrates regression of wall thickening and enhancement involving the right vertebral artery V4 segment ((**D**), arrow) and bilateral MCA M1 segments, with residual left-sided abnormality ((**E**), arrow). Follow-up diffusion-weighted imaging shows a lacunar sequela at the site of the previous pontine infarct ((**F**), arrow).

Seventeen patients underwent serial HR-VWI. VWE grade decreased in 13/17 patients (76.5%) and remained stable in 4/17 patients (23.5%); no patient showed an increased VWE grade. By follow-up interval, VWE grade decreased in 2/2 patients with follow-up of ≤90 days, 5/6 patients with follow-up of 91–365 days, 1/2 patients with follow-up of 366–730 days, and 5/7 patients with follow-up of >730 days. VWE grade remained stable in the remaining one, one, and two patients in the latter three strata, respectively, and no patient showed an increase. Because of the small stratum sizes, no interval-stratified hypothesis testing was performed. Interval-stratified findings are descriptive only and are summarized in Supplementary Tables S3–S5. A representative longitudinal case demonstrating diffuse left MCA M1 vessel wall enhancement with corresponding luminal narrowing on the initial examination and residual focal left ICA/proximal MCA enhancement on follow-up is shown in Figure 3. A second longitudinal example demonstrating initial multifocal involvement of the right vertebral artery V4 segment and bilateral MCA M1 segments, associated with an acute pontine infarct, and a subsequent reduction in vessel wall enhancement at follow-up is shown in Figure 4. Median VWE grade decreased from 3.0 (IQR, 2.0–3.0) at the first evaluable examination to 1.0 (IQR, 1.0–2.0) at the final evaluable examination. Maximum wall thickness decreased in 16/17 patients (94.1%) and remained stable in one patient (5.9%); no patient showed increased wall thickness. Median maximum wall thickness decreased from 2.0 mm (IQR, 1.8–2.8 mm) to 1.4 mm (IQR, 1.2–1.8 mm). Luminal narrowing grade decreased in seven patients, remained stable in ten, and increased in none. Median luminal narrowing grade was 3.0 (IQR, 1.0–3.0) at baseline and 2.0 (IQR, 0.0–3.0) at final follow-up. The principal imaging and longitudinal findings are summarized in Table 2. Patient-level VWE grade, VWE trajectory, maximum wall thickness, maximum stenosis grade, acute or subacute infarction status, and microbleed findings are provided in Supplementary Table S3.

## 4. Discussion

In this retrospective descriptive case series of patients evaluated for suspected inflammatory intracranial vasculopathy, HR-VWI demonstrated VWE in all 19 included patients, with a purely concentric pattern in 15 patients. The most frequently involved arteries were the MCA and ICA. MRI-defined acute or early subacute ischemic lesions were present in 13/19 patients. Among the 17 patients with serial imaging, VWE grade and maximum wall thickness decreased in most patients, whereas luminal narrowing was more frequently stable. These findings provide a descriptive account of HR-VWI morphology and longitudinal imaging changes in this selected cohort but do not establish diagnostic value or performance.

The predominance of concentric VWE in this cohort is broadly consistent with previous reports describing smooth concentric wall thickening and enhancement as a common HR-VWI feature of inflammatory intracranial vasculopathy. However, the proportion of purely concentric enhancement in our cohort, 15/19 cases (78.9%), was somewhat lower than previously reported rates, including 88.3% in the study by Patzig et al. and 92.3% in the study by D’Aniello et al. [13,19]. This difference may reflect the real-world composition of our cohort, which included patients evaluated for suspected inflammatory intracranial vasculopathy rather than only pathologically or clinically definite CNS vasculitis, as well as heterogeneity between primary and secondary forms of CNS vasculitis. In addition, our case-level classification separated purely concentric enhancement from mixed or asymmetric patterns, which may have lowered the proportion categorized as purely concentric. Therefore, concentric enhancement was frequently observed in this selected cohort, but this pattern should not be regarded as specific for CNS vasculitis. Mixed or eccentric components were also present, and VWE morphology must be interpreted together with clinical, laboratory, luminal, and longitudinal findings.

The frequent involvement of the MCA and ICA in our cohort is compatible with the known visibility and clinical relevance of medium- and large-sized intracranial arteries on 3T HR-VWI. Although prior studies have not uniformly reported vessel involvement using identical arterial-based categories, similar anterior-circulation predominance has been described. D’Aniello et al. reported anterior circulation involvement in most patients with confirmed CNS vasculitis, and Yang et al. similarly observed predominant anterior circulation involvement in their HR-MRI cohort [19,20]. Patzig et al. also emphasized medium-/large-vessel CNS vasculitis and found that diffusion-restricted lesions were more frequently associated with stenoses showing vessel wall contrast enhancement [13]. In our cohort, MCA and ICA predominance may therefore reflect both disease distribution and the greater reliability of HR-VWI assessment in proximal and medium-sized intracranial arteries. Although enhancement and infarction frequently coexisted, the small and diagnostically heterogeneous cohort was not suitable for reliable association testing. The descriptively similar luminal-narrowing grades observed in patients with and without MRI-defined acute or early subacute ischemic lesions cannot establish or exclude an association between stenosis severity and these imaging findings. Therefore, causal inference cannot be made from this descriptive case series.

An important observation in the present study was the longitudinal reduction in both VWE grade and measured maximum wall thickness. Among the 17 patients with serial HR-VWI, VWE grade decreased in 13 patients and remained stable in four, whereas maximum wall thickness decreased in 16 patients and remained stable in one. Luminal-narrowing grades were decreased in seven but remained stable in ten. The observed evolution of VWE is broadly consistent with prior longitudinal HR-VWI studies. Obusez et al. reported that, among CNS vasculitis patients with follow-up imaging, some showed resolution of vessel wall abnormalities while others had stable persistent enhancement; by contrast, most reversible cerebral vasoconstriction syndrome (RCVS) patients showed resolution of wall thickening and enhancement on follow-up [21]. Patzig et al. also demonstrated that regression or absence of VWE was associated with fewer relapses, whereas stable or progressive enhancement was more frequently associated with relapse; however, they emphasized that persistent enhancement may occur despite immunosuppressive therapy and clinical remission [13]. Because treatment timing, clinical response, and disease activity measures were incompletely available, these imaging changes cannot be interpreted as direct evidence of treatment response or active vascular inflammation.

The role of HR-VWI should be interpreted within the broader diagnostic framework of CNS vasculitis. Conventional luminal imaging may demonstrate stenosis, occlusion, or multifocal irregularity, but these findings are nonspecific and overlap with RCVS, intracranial atherosclerosis, dissection, moyamoya vasculopathy, and other inflammatory or infectious vasculopathies. Vessel wall MRI adds value by directly evaluating the arterial wall, but the presence of enhancement alone is insufficient for diagnosis. Patzig et al. explicitly noted that VWE is not exclusive to vasculitis and can occur in several mimics, including atherosclerosis, moyamoya vasculopathy, and RCVS [13]. Similar diagnostic uncertainty has been recognized in previous HR-VWI cohorts, in which case classification often relied on integrated clinical, laboratory, angiographic, and follow-up assessment rather than histopathological confirmation alone [13,17]. In the present study, HR-VWI morphology contributed both to multidisciplinary clinical assessment and to the imaging findings subsequently described. Therefore, selection and incorporation bias cannot be excluded. In particular, the observation of VWE in 19/19 included patients should not be interpreted as the prevalence of VWE among patients with CNS vasculitis or as an estimate of diagnostic sensitivity, specificity, or accuracy. Accordingly, the findings of the present study should be considered supportive rather than diagnostic.

This study has several limitations. First, the study was retrospective and descriptive, with a relatively small sample size, reflecting the rarity of CNS vasculitis and the real-world challenges of data collection. Because patients underwent HR-VWI based on clinical suspicion and referral patterns, referral and selection bias are possible, and the cohort may overrepresent patients with more conspicuous or persistent vascular abnormalities. Second, although all patients received treatment during their clinical course, treatment type, dose, duration, exact timing relative to HR-VWI examinations, treatment modifications, clinical response, relapse status, and disability outcomes could not be consistently retrieved. Consequently, the observed longitudinal imaging changes cannot be interpreted as natural history, therapeutic response, disease control, or change in inflammatory activity, and reliable treatment–imaging and clinical–imaging correlations could not be performed. Third, follow-up intervals were not standardized and ranged from 15 to 4034 days. The first-versus-final comparison therefore combines imaging changes observed over markedly different intervals and cannot be interpreted as representing a common biological time course. Although the results were stratified descriptively by clinically meaningful follow-up intervals, the small number of patients within each stratum precluded reliable interval-specific inference or hypothesis testing. Fourth, the cohort was diagnostically heterogeneous and included both primary and secondary forms of CNS vasculitis, which may have contributed to variability in the imaging findings and comprised seven patients with an established clinical diagnosis and 12 patients with clinical suspicion without definitive confirmation. The cohort also combined pediatric and adult patients, including four patients younger than 18 years. Childhood inflammatory CNS vasculopathies may differ from adult disease in diagnostic frameworks, etiologic distribution, relevant mimics, and clinical management. A uniform pediatric-specific diagnostic framework had not been prospectively applied and could not be reliably reconstructed from the available records. Although the adult-only sensitivity description showed findings similar to those of the full cohort, the small number of pediatric patients precluded meaningful age-stratified comparisons, and residual age-related heterogeneity remains an important limitation. Furthermore, histopathological confirmation was available in only a minority of patients, and most cases were classified through multidisciplinary assessment based on clinical, laboratory, conventional imaging, HR-VWI, and follow-up findings. Although this reflects real-world practice, it limits definitive etiological classification. Because HR-VWI contributed to the consensus diagnosis, incorporation bias cannot be excluded and may have increased the apparent frequency and reduced the estimated specificity of the observed imaging patterns. The longitudinal measurements were based on the maximum abnormality identified independently at each examination rather than on mandatory segment-matched measurements. The segment contributing the maximum value could therefore differ between baseline and follow-up. Images were not formally coregistered, and no dedicated correction for partial-volume effects or technical repeatability assessment was performed. Consequently, the observed changes may partly reflect lesion selection and measurement variability and should not be interpreted as precise segment-specific regression. In addition, all examinations were reviewed jointly in consensus, with baseline and follow-up studies evaluated together and examination order known to the readers. Independent, deidentified, randomly ordered readings were not performed, and interobserver agreement could not be calculated. No control group comprising alternative intracranial vasculopathies was included because the study was designed as a descriptive case series rather than a diagnostic-accuracy study. Consequently, the specificity of the observed HR-VWI patterns and their ability to distinguish CNS vasculitis from relevant mimics could not be assessed. The small sample size also precluded reliable hypothesis testing, subgroup comparisons, and multivariable modelling; consequently, the findings should be regarded as descriptive and hypothesis-generating.

Future studies should aim to validate these findings in larger multicentric cohort studies, with structured follow-up. Inclusion of relevant control groups would allow assessment of the incremental diagnostic value of HR-VWI over conventional MRI, MRA, CTA, and DSA.

## 5. Conclusions

In this selected retrospective case series of patients evaluated for suspected inflammatory intracranial vasculopathy, concentric VWE was observed in 15/19 patients, with the MCA and ICA being the most frequently involved arteries. The main contribution of this study is the descriptive longitudinal assessment of serial HR-VWI examinations in a real-world cohort. VWE grade and vessel wall thickness decreased in most patients with serial imaging, whereas luminal narrowing was more frequently stable. Because treatment timing, clinical response, and disease activity measures were incompletely available, these imaging changes cannot be interpreted as direct evidence of treatment response or active vascular inflammation. HR-VWI may provide complementary morphologic and longitudinal information, but persistent enhancement, wall thickening, and variable follow-up findings require cautious interpretation together with the clinical, laboratory, and luminal imaging findings. Prospective studies incorporating standardized treatment information, clinical outcomes, relapse assessment, and predefined imaging intervals are needed to determine whether longitudinal VWE and vessel wall thickness changes correlate with inflammatory activity or therapeutic response.

## Figures and Tables

**Table 1:** Case-level summary of included patients.

Age/Sex	Clinical Diagnosis/Status	No. Exams	Follow-Up Days	Main Arteries Involved	VWE Pattern	Max VWE Grade	VWE Trajectory	Max Wall Thickness	Max Stenosis Grade	MRI-Defined Acute or Early Subacute Ischemic Lesion	Microbleeds
79/M	Clinical Suspicion	2	15	Left PCA, MCA	Concentric	3	Decreased	2.0 mm	1	No	No
68/F	Sjögren	1	—	Right MCA	Concentric	3	Single exam	1.8 mm	1	Yes	Yes
6/F	CNS vasculitis	4	96	Bilateral ICA, MCA	Concentric	3	Decreased	3.5 mm	3	Yes	Yes
66/F	Sjögren	2	116	Left ICA, bilateral MCA, left PCA	Mixed concentric/eccentric	3	Decreased	2.8 mm	0	Yes	No
32/F	Clinical Suspicion	3	475	Right ICA, MCA	Concentric	1	Stable	3.0 mm	0	No	No
41/M	CNS vasculitis	6	4034	Right MCA	Concentric	3	Decreased	2.0 mm	1	No	Yes
46/F	Clinical Suspicion	1	—	Right ICA	Concentric	2	Single exam	2.8 mm	1	Yes	No
52/F	Clinical Suspicion	4	1271	Vertebral artery, PCA, PICA	Concentric/mixed	3	Stable	2.8 mm	3	No	No
12/F	Clinical Suspicion	8	1520	ICA, MCA	Concentric	2	Decreased	1.4 mm	1	Yes	No
6/M	Clinical Suspicion	5	2708	ICA, MCA	Concentric	3	Decreased	2.5 mm	2	Yes	No
49/M	Clinical Suspicion	9	439	ICA, bilateral MCA	Concentric	3	Decreased	2.4 mm	3	Yes	Yes
42/F	Clinical Suspicion	4	2312	Left ICA, MCA	Concentric	2	Decreased	2.0 mm	2	Yes	No
15/F	Clinical Suspicion	2	427	Left ICA, bilateral MCA	Concentric	2	Stable	1.5 mm	2	No	Yes
49/M	Clinical Suspicion	5	1819	Bilateral MCA, PCA, basilar, VA	Concentric	2	Stable	1.6 mm	2	Yes	Yes
39/M	Takayasu	10	2009	VA, PCA, MCA, carotid arteries	Concentric	3	Decreased	3.8 mm	0	Yes	Yes
40/F	Clinical Suspicion	7	383	Left ICA, MCA	Eccentric/mixed	2	Decreased	1.8 mm	3	Yes	No
61/M	Primary CNS vasculitis	6	730	PCA, VA, ICA, MCA	Concentric/asymmetric	3	Decreased	2.2 mm	3	Yes	Yes
59/M	Clinical Suspicion	5	26	VA, basilar, MCA, PICA	Concentric	2	Decreased	1.8 mm	1	Yes	No
71/F	Rheumatoid arthritis	3	391	MCA, ICA, basilar	Concentric/no residual abnormality on follow-up	3	Decreased	3.4 mm	3	No	Yes

Follow-up duration was not applicable to patients with a single examination. HR-VWI, high-resolution vessel wall imaging; ICA, internal carotid artery; MCA, middle cerebral artery; PCA, posterior cerebral artery; PICA, posterior inferior cerebellar artery; VA, vertebral artery; VWE, vessel wall enhancement.

**Table 2:** Imaging and longitudinal findings.

Domain	Finding	Result
Vessel wall enhancement	VWE present	19/19 (100%)
	Purely concentric pattern	15/19 (78.9%)
	Mixed or asymmetric pattern	4/19 (21.1%)
Arteries involved	Middle cerebral artery	17/19 (89.5%)
	Internal carotid artery	13/19 (68.4%)
	Posterior cerebral artery	6/19 (31.6%)
	Vertebral artery	5/19 (26.3%)
	Basilar artery	3/19 (15.8%)
	Posterior inferior cerebellar artery	2/19 (10.5%)
Parenchymal and hemorrhagic findings	Acute or subacute infarction	13/19 (68.4%)
	Chronic infarct or encephalomalacia	14/19 (73.7%)
	Microbleeds	9/19 (47.4%)
	Organized intracranial hematoma	1/19 (5.3%)
Longitudinal findings	Serial imaging available	17/19 (89.5%)
	VWE grade: decreased/stable/increased	13/4/0
	VWE grade, baseline to final	3.0 to 1.0
	Wall thickness: decreased/stable/increased	16/1/0
	Wall thickness, baseline to final	2.0 to 1.4 mm
	Luminal narrowing: decreased/stable/increased	7/10/0
	Luminal narrowing grade, baseline to final	3.0 to 2.0

Data are presented as n/N (%) or counts unless otherwise indicated. Vascular territories and associated imaging findings were not mutually exclusive. Longitudinal changes were evaluated in the 17 patients with serial imaging. VWE, vessel wall enhancement.

## Data Availability

The datasets generated during and/or analyzed during the current study are available from the corresponding author on reasonable request. The data are not publicly available because they contain detailed clinical and imaging information from a small retrospective cohort, and public release could compromise participant confidentiality. Requests for access will be considered in accordance with applicable institutional and ethical data-protection requirements.

## Supplementary Materials

The following supporting information can be downloaded at https://www.mdpi.com/article/10.3390/tomography12080116/s1, Table S1: Brain MRI acquisition parameters for the 3T intracranial vessel wall MRI protocol—High-resolution vessel wall and angiographic sequences; Table S2: Brain MRI acquisition parameters for the 3T intracranial vessel wall MRI protocol—Conventional parenchymal sequences; Figure S1: Representative vessel wall measurement methodology; Table S3: Patient-level cohort and follow-up characteristics; Supplementary Table S4: Patient-level HR-VWI severity and associated imaging findings; Supplementary Table S5: Descriptive stratification of VWE-grade trajectories by follow-up interval.

## Abbreviations

The following abbreviations are used in this manuscript:

ADC	apparent diffusion coefficient
ANCA	antineutrophil cytoplasmic antibody
CI	confidence interval
CNS	central nervous system
CNSV	central nervous system vasculitis
CTA	computed tomography angiography
DSA	digital subtraction angiography
DWI	diffusion-weighted imaging
FLAIR	fluid-attenuated inversion recovery
HR-VWI	high-resolution vessel wall imaging
ICA	internal carotid artery
IQR	interquartile range
MCA	middle cerebral artery
MRA	magnetic resonance angiography
MRI	magnetic resonance imaging
PACNS	primary angiitis of the central nervous system
PCA	posterior cerebral artery
PICA	posterior inferior cerebellar artery
RCVS	reversible cerebral vasoconstriction syndrome
SLE	systemic lupus erythematosus
SWI	susceptibility-weighted imaging
VA	vertebral artery
VWE	vessel wall enhancement
