# Supplementary material for: High-Resolution 3T Intracranial Vessel Wall MRI in Patients Evaluated for Suspected Inflammatory Intracranial Vasculopathy: Morphologic and Follow-Up Findings from a Retrospective Case Series
Source: Tomography. 2026 Aug 19;12(8):116. doi: 10.3390/tomography12080116 (PMC13517854; doi:10.3390/tomography12080116)
Supplement: Supplementary file 1 [file tomography-12-00116-s001.zip › tomography-4477686-supplementary.pdf]

Supplementary Materials

Scanner platform: Siemens MAGNETOM 3.0T XT, Numaris/X VA60A-0D4N. The intracranial vessel-wall sequence was a sagittal 3D variable-flip-angle turbo spin-echo SPACE fat-suppressed black-blood acquisition performed before and after contrast administration. Coil selection was automatic for all listed acquisitions.

Table S1. Brain MRI acquisition parameters for the 3T intracranial vessel wall MRI protocol—High-resolution vessel wall and angiographic sequences.

Parameters	Precontrast 3D T1 SPACE FS	Postcontrast 3D T1 SPACE FS	3D T2 SPACE dark-fluid	3D TOF MRA	Dynamic TWIST MRA
**Acquisition time**	8:57 min	8:57 min	4:28 min	6:44 min	59 sec
**Voxel size (mm3)**	0.6 x 0.6 x 0.6	0.6 x 0.6 x 0.6	0.8 x 0.8 x 0.9	0.3 x 0.3 x 0.4	0.9 x 0.9 x 1.0
**Plane / orientation**	Sagittal	Sagittal	Sagittal	Transversal/oblique	Coronal
**Slabs or slices**	1 slab; 224 slices/slab	1 slab; 224 slices/slab	1 slab; 192 slices/slab	4 slabs; 72 slices/slab	1 slab; 120 slices/slab
**FOV read (mm)**	220	220	240	200	230
**FOV phase (%)**	100.0	100.0	100.0	90.6	81.3
**Slice thickness (mm)**	0.60	0.60	0.90	0.40	1.00
**TR (ms)**	900.0	900.0	7000.0	21.10	2.34
**TE (ms)**	15.00	15.00	395.00	3.69	0.98
**TI / preparation**	-	-	TI 2050 ms; non-selective T2-prep IR; T2-prep duration 125 ms	-	-
**Flip angle / mode**	T1 variable	T1 variable	T2 variable	20 deg	18 deg
**Fat suppression**	Strong fat saturation	Strong fat saturation	Strong fat saturation	Standard	Standard
**Base resolution**	384	384	288	384	256
**Phase resolution (%)**	100	100	90	99	70
**Slice resolution (%)**	100	100	90	66	75
**Acceleration mode**	GRAPPA	GRAPPA	Compressed sensing	Compressed sensing	GRAPPA
**Total acceleration factor**	2	2	5.0	4.4	6
**Acceleration details**	PE 2; 3D 1	PE 2; 3D 1	PE reference lines 45; 3D reference lines 24	Reference lines PE 24; 3D 24	PE 2; 3D 3
**Partial Fourier / echo**	Slice partial Fourier 6/8	Slice partial Fourier 6/8	-	Asymmetric echo weak	Phase 6/8; slice 6/8; asymmetric echo strong
**Trajectory / reordering**	Radial	Radial	Linear	Cartesian; linear	TWIST; symmetric share
**Bandwidth (Hz/Px)**	449	449	643	186	750
**Echo spacing (ms)**	5.04	5.04	3.76	9.58	-
**Turbo factor / EPI factor**	63	63	220	-	-
**Other sequence-specific settings**	Elliptical scanning on	Elliptical scanning on	Elliptical scanning on	Flow direction F >> H; TONE ramp 70%; flow compensation slice/read	Central region A 30%; sampling density B 20%; 16 measurements; temporal resolution 2.98 s; time to center 8.6 s

Table S2. Brain MRI acquisition parameters for the 3T intracranial vessel wall MRI protocol—Conventional parenchymal sequences.

Parameters	Axial T2 TSE	3D SWI	RESOLVE DWI
**Acquisition time**	2:16 min	3:03 min	1:31 min
**Voxel size (mm3)**	0.2 x 0.2 x 5.0	0.6 x 0.6 x 2.0	0.5 x 0.5 x 4.5
**Plane / orientation**	Transversal/oblique	Transversal	Transversal/oblique
**Slices or slabs**	30 slices	1 slab; 72 slices/slab	30 slices
**Distance factor (%)**	10	20	20
**FOV read (mm)**	220	220	220
**FOV phase (%)**	81.3	87.5	100.0
**Slice thickness (mm)**	5.0	2.00	4.5
**TR (ms)**	6250.0	30.0	1900.0
**TE (ms)**	111.00	19.80	TE1 65; TE2 113
**Flip angle**	150 deg	15 deg	180 deg
**Fat suppression**	Standard	Standard	Strong fat saturation
**Base resolution**	512	368	224
**Phase resolution (%)**	70	100	80
**Slice resolution (%)**	-	100	-
**Acceleration mode**	GRAPPA	Wave-CAIPI	SMS

Parameters	Axial T2 TSE	3D SWI	RESOLVE DWI
**Acceleration details**	PE factor 4; reference lines PE 32; Deep Resolve on	PE factor 2; 3D factor 2; reference lines PE 30 and 3D 30	PE factor 2; SMS factor 3; FOV shift factor 2
**Partial Fourier / readout**	-	-	Readout partial Fourier 7/8; readout segments 7
**Bandwidth (Hz/Px)**	444	100	558
**Echo spacing (ms)**	11.1	-	0.40
**Turbo factor / EPI factor**	Turbo factor 18; 5 echo trains/slice	-	EPI factor 90
**Diffusion settings**	-	-	4-scan trace; 4 directions; b = 0 and 1000 s/mm2; calculated b = 2000 s/mm2; ADC maps on
**Other sequence-specific settings**	Averages 4; interleaved acquisition	SWI on; magnitude/phase reconstruction; flow compensation on	Trace-weighted images on; reacquisition mode on

The postcontrast 3D T1 SPACE FS acquisition was initiated immediately after intravenous gadolinium administration; the exact postcontrast delay in minutes was not separately recorded. ADC = apparent diffusion coefficient; CS = compressed sensing; DWI = diffusion-weighted imaging; FOV = field of view; FS = fat-saturated; GRAPPA = generalized autocalibrating partially parallel acquisitions; IR = inversion recovery; MRA = magnetic resonance angiography; PE = phase encoding; SPACE = Sampling Perfection with Application-optimized Contrasts using different flip-angle Evolutions; SWI = susceptibility-weighted imaging; TE = echo time; TI = inversion time; TOF = time-of-flight; TR = repetition time; TSE = turbo spin echo; TWIST = time-resolved angiography with stochastic trajectories.

Supplementary Table S3. Patient-level cohort and follow-up characteristics.

Study ID	Age/Sex	Clinical diagnosis/status	No. exams	Follow-up days	Follow-up interval	Main arteries involved	VWE pattern
P01	79/M	Clinical Suspicion	2	15	≤90 days	Left PCA, MCA	Concentric
P02	68/F	Sjögren	1	0	Single examination	Right MCA	Concentric
P03	6/F	CNS vasculitis	4	96	91–365 days	Bilateral ICA, MCA	Concentric
P04	66/F	Sjögren	2	116	91–365 days	Left ICA, bilateral MCA, left PCA	Mixed concentric/eccentric
P05	32/F	Clinical Suspicion	3	237	91–365 days	Right ICA, MCA	Concentric
P06	41/M	CNS vasculitis	6	4034	>730 days	Right MCA	Concentric
P07	46/F	Clinical Suspicion	1	0	Single examination	Right ICA	Concentric
P08	52/F	Clinical Suspicion	4	879	>730 days	Vertebral artery, PCA, PICA	Concentric/mixed
P09	12/F	Clinical Suspicion	8	1520	>730 days	ICA, MCA	Concentric
P10	6/M	Clinical Suspicion	5	2708	>730 days	ICA, MCA	Concentric
P11	49/M	Clinical Suspicion	9	356	91–365 days	ICA, bilateral MCA	Concentric
P12	42/F	Clinical Suspicion	4	2312	>730 days	Left ICA, MCA	Concentric
P13	15/F	Clinical Suspicion	2	427	366–730 days	Left ICA, bilateral MCA	Concentric
P14	49/M	Clinical Suspicion	5	1819	>730 days	Bilateral MCA, PCA, basilar, VA	Concentric
P15	39/M	Takayasu	10	2009	>730 days	VA, PCA, MCA, carotid arteries	Concentric
P16	40/F	Clinical Suspicion	7	329	91–365 days	Left ICA, MCA	Eccentric/mixed
P17	61/M	Primary CNS vasculitis	6	290	91–365 days	PCA, VA, ICA, MCA	Concentric/asymmetric
P18	59/M	Clinical Suspicion	5	21	≤90 days	VA, basilar, MCA, PICA	Concentric
P19	71/F	Rheumatoid arthritis	3	391	366–730 days	MCA, ICA, basilar	Concentric/no residual abnormality on follow-up

Note: Follow-up days represent the interval between the first and final evaluable HR-VWI examinations. A value of 0 indicates a single examination. ICA, internal carotid artery; MCA, middle cerebral artery; PCA, posterior cerebral artery; PICA, posterior inferior cerebellar artery; VA, vertebral artery; VWE, vessel wall enhancement.

Supplementary Table S4. Patient-level HR-VWI severity and associated imaging findings.

Study ID	Max VWE grade	VWE trajectory	Max wall thickness	Max stenosis grade	Acute/subacute infarct inferred	Microbleeds
P01	3	Decreased	2.0 mm	1	No	No
P02	3	Single exam	1.8 mm	1	Yes	Yes
P03	3	Decreased	3.5 mm	3	Yes	Yes
P04	3	Decreased	2.8 mm	0	Yes	No
P05	1	Stable	3.0 mm	0	No	No
P06	3	Decreased	2.0 mm	1	No	Yes
P07	2	Single exam	2.8 mm	1	Yes	No
P08	3	Stable	2.8 mm	3	No	No
P09	2	Decreased	1.4 mm	1	Yes	No
P10	3	Decreased	2.5 mm	2	Yes	No
P11	3	Decreased	2.4 mm	3	Yes	Yes
P12	2	Decreased	2.0 mm	2	Yes	No
P13	2	Stable	1.5 mm	2	No	Yes
P14	2	Stable	1.6 mm	2	Yes	Yes
P15	3	Decreased	3.8 mm	0	Yes	Yes
P16	2	Decreased	1.8 mm	3	Yes	No
P17	3	Decreased	2.2 mm	3	Yes	Yes
P18	2	Decreased	1.8 mm	1	Yes	No
P19	3	Decreased	3.4 mm	3	No	Yes

Note: Maximum VWE grade, wall thickness, and stenosis grade are patient-level per-examination summary measures. Longitudinal VWE trajectory compares the first and final evaluable examinations. VWE, vessel wall enhancement.

Supplementary Table S5. Descriptive stratification of VWE-grade trajectories by follow-up interval.

Follow-up interval	Patients, n	VWE grade decreased, n	VWE grade stable, n	VWE grade increased, n
≤90 days	2	2	0	0
91–365 days	6	5	1	0
366–730 days	2	1	1	0
>730 days	7	5	2	0

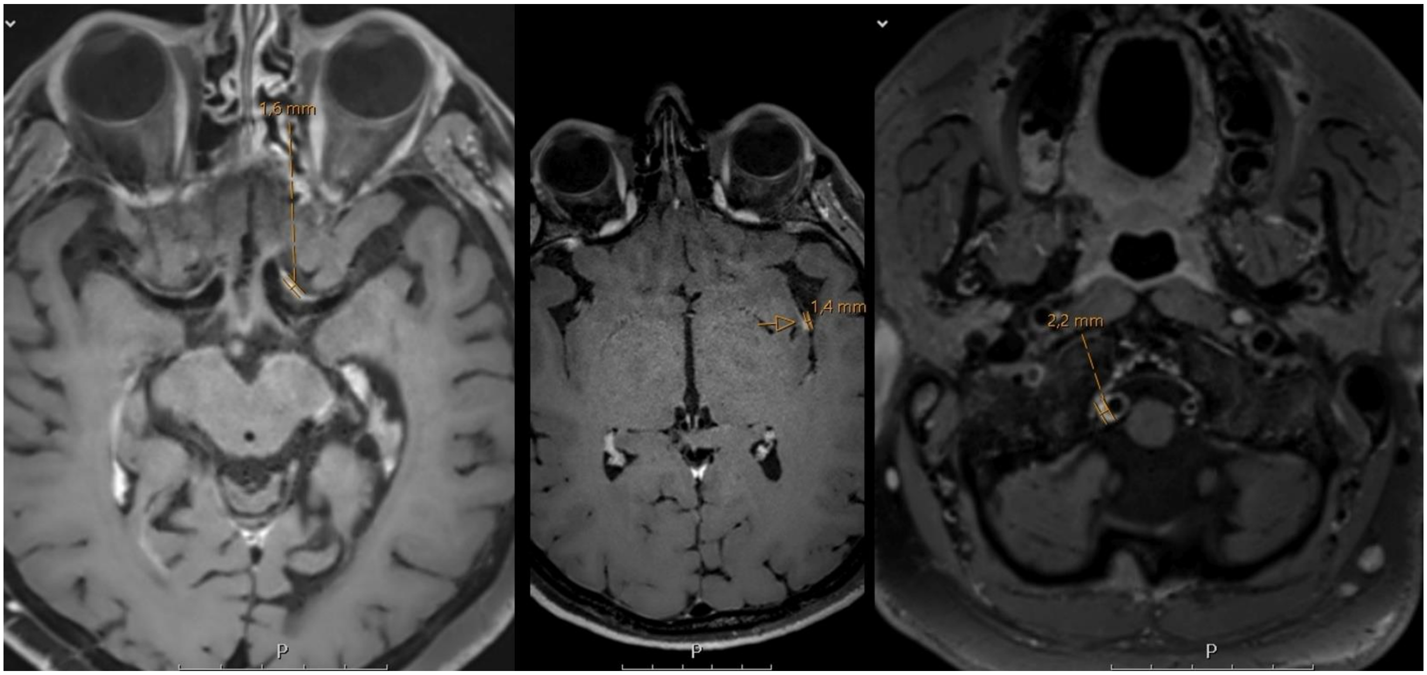

Figure S1: Representative vessel wall measurement methodology.
